# Masculine Identity, Body Image and Illness-Related Shame: Pathways to Psychological Distress in Men with Fibromyalgia

**DOI:** 10.3390/healthcare14050606

**Published:** 2026-02-27

**Authors:** Shulamit Geller, Sigal Levy, Ronit Avitsur

**Affiliations:** 1 School of Behavioral Sciences, The Academic College of Tel Aviv-Yaffo, Tel Aviv-Yaffo 6818211, Israel; avitsur@mta.ac.il; 2 Statistics Education Unit, The Academic College of Tel Aviv-Yaffo, Tel Aviv-Yaffo 6818211, Israel; levy@mta.ac.il

**Keywords:** fibromyalgia, masculine identity, body appreciation, illness-related shame, psychological distress

## Abstract

Objective: Although recognition of fibromyalgia (FMS) in men is growing, the mechanisms that contribute to their psychological distress remain poorly understood. This study aims to clarify how FMS alters men’s psychological distress and to identify potential protective and risk factors involved in this process in this often-underrepresented population. Methods: This study comprised a total of 225 men aged 18–75; of these, 102 were men with FMS (based on self-report) and 123 were healthy peers (HPs), all of whom completed questionnaires on demographics, anxiety (GAD-7), depression (PHQ-9), body appreciation (BAS-2), masculine self-esteem (MSES), illness-related shame (CISS), and pain intensity (SF-MPQ). Results: Men with FMS reported significantly higher depression and anxiety, lower body appreciation, and compromised masculine identity. Between-group analysis showed body appreciation mediated the fibromyalgia–distress relationship. However, within the FMS group, compromised masculine identity and illness-related shame were the strongest pathways to distress, while body appreciation showed no effect. Moderation analysis confirmed body appreciation buffered distress in controls but not in men with FMS. Conclusion: Masculine identity threats and illness-related shame constitute central mechanisms of psychological distress in men with FMS. Body appreciation operates differently in this population than in healthy men. Findings underscore the need for gender-sensitive interventions addressing identity disruption and emphasizing functionality over appearance-based acceptance.

## 1. Introduction

Fibromyalgia syndrome is a chronic condition involving persistent, widespread pain with associated fatigue, stiffness, sleep disturbance, and functional impairment [1]. Anxiety and depression are highly prevalent and often exceed rates found in other chronic pain conditions and healthy populations [2]. Although reports estimated female-to-male ratio of FMS prevalence at 3:1 [3], growing evidence indicates that FMS is underdiagnosed in men due to diagnostic bias [4]. Gender norms and patterns of symptom reporting further widen this gap [5], placing men in what Conversano et al. [6] describe as a “double burden of credibility,” linked to both a contested diagnosis and the stigma of a condition perceived as feminine. This contributes to clinical skepticism [7], causing diagnostic delays, self-doubt [8], and reduced access to treatment, thus producing psychological consequences that remain insufficiently understood in men with FMS [9].

Men with fibromyalgia exhibit high anxiety and depression scores [10], often experiencing equal or greater psychological distress (PD) than women. Henao-Pérez et al. [11] identified male sex as a distinct risk factor, with depression and anxiety present in 42.6% of men compared to 28% of women with FMS. Contributing factors include the condition’s invisibility, which generates both a credibility and identity crisis that challenges masculine self-concept [12]; cognitive dysfunction that undermines competence and productivity [6]; loss of functional capacity in work and physical activities [9]; and sexual dysfunction, which further threatens self-esteem and intimate relationships [6].

In studies of women with FMS, evidence shows that while it is directly associated with PD, indirect mechanisms also contribute to this relationship [13,14]. Specifically, self-compassion and body appreciation were identified as protective factors against PD among women with FMS [14], while social comparison strategies mediated the link between FMS and PD [13]. The present study aims to develop an explanatory model for the effect of FMS on men’s PD, identifying the interplay between body appreciation, illness-related shame, and masculinity.

### 1.1. Body Image

Body image is a multifaceted psychological construct encompassing individuals’ perceptions and attitudes toward their bodies, including associated thoughts, beliefs, emotions, and behaviors [15]. This notion has received increasing attention in health-related research, with growing recognition of the importance of incorporating the embodied experiences, particularly experiences of pain and disability, into the understanding of body image [16].

One important dimension of body image is body appreciation, a positive aspect that refers to the unconditional acceptance, respect, and favorable evaluation of one’s body [17]. Body appreciation has been identified as a potential protective factor in the psychological wellbeing of individuals with chronic illnesses. For instance, across patient samples, greater body appreciation has been associated with better quality of life [18,19].

Women with fibromyalgia frequently report disturbed body image, including distrust of the body, functional limitations, reduced vitality, and weight concerns [20,21]. Even without visible deformity, the condition is consistently linked to negative perceptions of appearance and functionality and to heightened PD [21,22]. Pain severity is closely tied to poorer body appreciation, shaped by localized pain, cognitive symptoms, negative healthcare experiences, activity restrictions, and reduced quality of life [20,22]. Although women report slightly higher generalized pain and somatic symptoms, men show similar diagnostic profiles [4]. For men, fibromyalgia often triggers a sharp shift in bodily self-perception: from strength and reliability to vulnerability and loss of control [6,12]. This reflects both activity limitations [23] and a deeper rupture in bodily identity, with men describing their bodies as alien and untrustworthy [12]. Evidence on body appreciation in men with fibromyalgia is minimal. The only study testing gender as a moderator of the pain–body image link found no differences, suggesting similar mechanisms across genders [24]. More research is needed to determine whether body appreciation buffers PD in men with fibromyalgia.

### 1.2. Masculine Identity

Traditional masculine identity is closely tied to physical capability, including strength, endurance, functional competence, and sexual performance [25]. Because masculinity is a culturally contingent and often fragile construct [26], chronic illness can destabilize it. Fibromyalgia disrupts these embodied foundations, requiring what Conversano et al. [6] describe as an “inefficient renegotiation” of masculinity marked by perceived inadequacy. Loss of capacity for roles in physical labor, sport, and breadwinning weakens core identity anchors [12], a pattern also seen in other chronic conditions such as Lyme disease [27]. This threat extends beyond personal experience to societal perceptions: healthcare professionals and the public often view men with chronic pain as less masculine, attributing dependency and emotional vulnerability [28]. Given that fibromyalgia is culturally coded as a feminine condition, men face pressures to maintain provider roles and adhere to stoic norms that inhibit help seeking and emotional expression [7,29]. Many therefore avoid seeking help due to shame and perceived masculinity loss [6,12], increasing vulnerability to psychological distress across multiple domains.

### 1.3. Illness-Related Shame in FMS

Shame is an essential mechanism linking impaired body image to PD in men with fibromyalgia. Defined by global inadequacy and reduced self-worth [30], shame is strongly associated with depression and anxiety across chronic illness [31,32]. In chronic-pain populations, it can be more distressing than the pain itself [33], and shame proneness is markedly elevated compared to HPs [34]. Among men with fibromyalgia, shame arises through three interconnected pathways. Illness-related shame may arise when physical changes or symptoms are perceived as falling short of ideals of strength and capability. Both visible challenges and less apparent symptoms, such as sexual dysfunction or cognitive fog, can contribute to these feelings of shame [9]. Internalized stigma adds a second layer, as men identify with a doubly stigmatized category: chronic-pain patients and men with a condition culturally framed as feminine [6,35]. A third pathway, role performance shame, emerges when illness-related limitations undermine traditional roles as provider, protector, or sexually capable partner, eroding self-worth [23]. Disrupted body image may therefore contribute to the development of illness-related shame, which in turn can intensify disruptions in gender role expectations and lead to heightened levels of depression and anxiety, underscoring the need for gender-sensitive interventions.

### 1.4. Pain

Chronic and diffuse pain is the core symptom of FMS [1], and heightened perception of pain has been found associated with high levels of anxiety and depression [13]. Drawing on the developmental theory of embodiment and fibromyalgia-specific literature, lived pain experiences in FMS shape body image [24]. Threats to masculine identity and illness-related shame, both linked to pain experiences, may mediate the development of anxiety and depression in men with FMS [7,12].

Thus, the overarching goal of the current study is to identify the roles and the interplay between body appreciation, illness-related shame, masculine identity and pain, in the manifestation of PD in men with FMS.

Within this context, the hypotheses of our study are as follows:

**Hypothesis 1:**  *Group differences*

**H1a:**  *Men with FMS will present higher PD, compromised sense of masculinity, and lower body appreciation than HPs.*

**H1b:**  *PD will be positively correlated with loss of masculinity and negatively correlated with body appreciation. In turn, body appreciation will be negatively correlated with compromised masculinity.*

**Figure 1:**
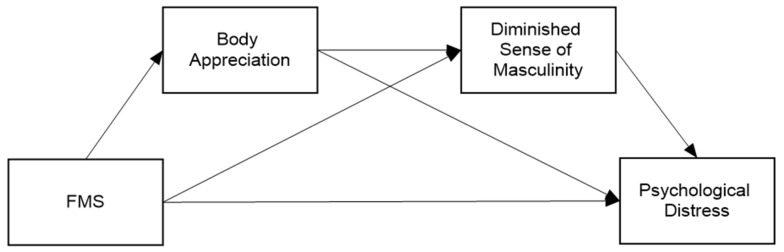
Hypothesized model for group comparison.

**H1c:**  *Body appreciation, followed by compromised masculinity, will mediate the link between FMS and PD (Figure 1).*

**Hypothesis 2:**  *Among men with FMS*

**H2a:**  *Illness characteristics—pain, illness-related shame, and compromised sense of masculinity—will be positively correlated with PD. Body appreciation will be negatively related to PD.*

**Figure 2:**
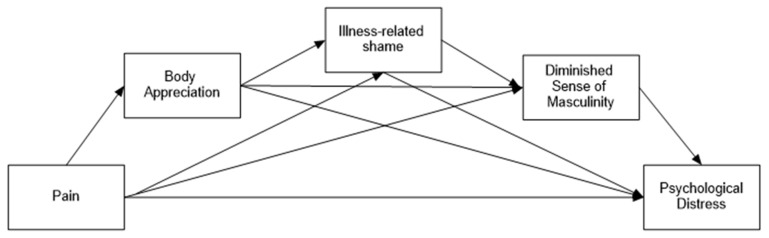
Hypothesized model among men with FMS.

**H2b:**  *Body appreciation, followed by illness-related shame and then compromised masculinity, will mediate the link between pain and PD (Figure 2).*

## 2. Materials and Methods

This cross-sectional survey was carried out in Israel from 2023 to 2024. Participants were recruited via relevant online forums and a snowball sampling, according to which research assistants approached potential participants among their acquaintances who, in turn, were asked to help recruit more participants. The study was approved by the Institutional Review Board (IRB) of The Academic College of Tel-Aviv Yaffo (# 2023315 on 16 December 2023). Participants provided informed consent and were assured that the questionnaire was completely anonymous and that they could discontinue participation at any time.

Inclusion criteria were men aged over 18 who were fluent in Hebrew. Control and experimental groups were defined using participants’ self-reported medical conditions, and diagnoses were not verified via medical records. The final sample included 348 men; of them, 123 (31%) were excluded due to missing data. The final sample comprised 225 men, of whom 102 (45%) had reported being diagnosed with FMS and 123 were HPs aged 18–75.

### 2.1. Measures

Participant background data included weight and height, age, marital status, and number of children. BMI was calculated using participants’ reported weight and height. Men who indicated a diagnosis of FMS also reported how long they had been living with the condition.

Depressive symptoms were measured with the 9-item Patient Health Questionnaire (PHQ-9) [36,37]. Each item ranges from 0 (not at all) to 3 (nearly every day). All scores are added together to obtain a global score, which ranges from 0 to 27, with higher scores indicating higher levels of depression. Internal consistency of the PHQ-9 in the current study was satisfactory (ω = 0.91).

Generalized anxiety was assessed using the 7-item Generalized Anxiety Disorder Scale (GAD-7) [38,39]. Items are rated from 0 (not at all) to 3 (nearly every day) and added together to yield a total score ranging from 0 to 21, with higher scores indicating higher levels of anxiety; internal consistency in the current study was satisfactory (ω = 0.93).

The Body Appreciation Scale-2 (BAS-2) [17,40] is a 10-item measure assessing acceptance, respect, and care for one’s body, as well as protection from unrealistic beauty standards. Items are rated from 1 (never) to 5 (always) and averaged to create a mean score, with higher values indicating greater body appreciation (ω = 0.93).

The Masculine Self-Esteem Scale (MSES) [41] is a self-report questionnaire for adult men assessing perceptions of masculinity. It includes eight items rated on a scale from 1 (strongly disagree) to 5 (strongly agree). Higher scores indicate lower masculinity self-esteem. Translated into Hebrew using the back-translation method, it showed satisfactory internal consistency in this study (ω = 0.93).

The Chronic-Illness-Related Shame Scale (CISS) [42] is a seven-item self-report measure assessing shame related to chronic illness. Items are rated on a Likert scale from 0 (not at all true) to 4 (very true), with higher scores indicating greater shame. Translated into Hebrew using the back-translation method [43], it showed satisfactory internal consistency in this study (ω = 0.88).

The Short-Form McGill Pain Questionnaire (SF-MPQ) [44,45] is a 15-item adjective checklist assessing perceived pain intensity, with items rated from 0 (none) to 3 (severe), and includes two single-item measures of present pain. For this study, the two present-pain items were combined, with higher scores indicating greater pain perception; internal consistency in the current sample was satisfactory (ω = 0.98).

### 2.2. Statistical Analysis

Descriptive statistics are presented as M (SD) for numeric measures and N (%) for categorical ones. Group differences were tested using one-way ANOVA and Chi-square tests. Pearson correlations were calculated between the main study variables. Process model 6 [46] was used to test hypotheses H1c and H2b, and model 1was used to test the follow-up moderation model. Power analysis was performed using G*Power 3.1.9.4. Results showed that the sample size used was sufficient for detecting a medium effect (f^2^ = 0.15) with a probability of more than 99%.

## 3. Results

Analysis of sample demographics showed that men with FMS are older, less educated, and less likely to be in relationships than the control group (Table 1). These background variables correlate with depression, and all but relationship status correlate with anxiety (Table 2). Of them, age will be controlled for in all further analysis, as it correlates with the other covariates. However, including it as a covariate yielded essentially similar results to those obtained when no covariate was included.

### 3.1. Hypothesis 1: Group Differences

- Hypothesis 1a

In accordance with hypothesis 1a, the results indicate that men with FMS reported on average more symptoms of anxiety and depression, lower body appreciation and compromised masculinity (Table 3).

- Hypothesis 1b

Table 4 indicates significant correlations among all primary study variables. The hypothesis was thus supported by the data.

- Hypothesis 1c

**Figure 3:**
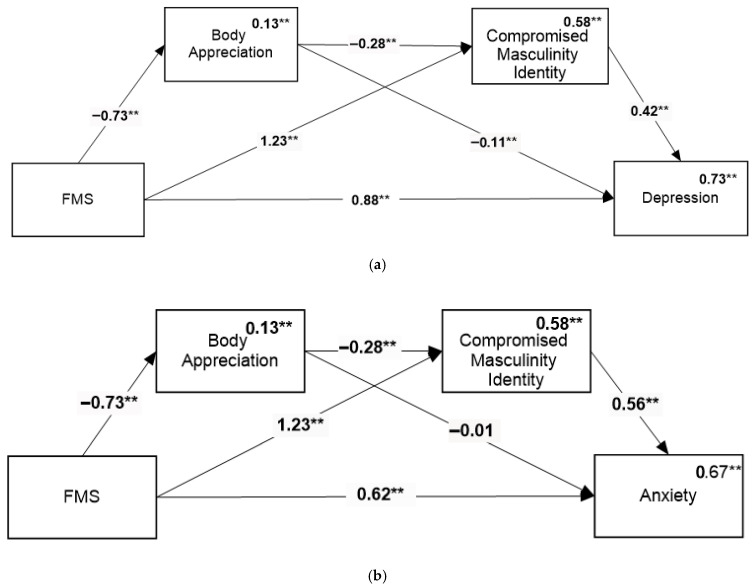
Mediation model for testing the relationship between FMS and depression (**a**) and the relationship between FMS and anxiety (**b**). Numbers above the lines are standardized path coefficients. Numbers above variable names are multiple squared correlations. ** *p* < 0.01.

In predicting depression (Figure 3a), we found that all direct paths were significant, as well as three indirect paths: through body appreciation (Beta = 0.08, 95%CI = [0.02, 0.16]), through compromised masculine identity (Beta = 0.52, 95%CI = [0.34, 0.70]), and through body appreciation followed by compromised masculine identity (Beta = 0.09, 95%CI = [0.04, 0.15]).

In predicting anxiety (Figure 3b), we found that all direct paths were significant except for the direct path between body appreciation and anxiety. Additionally, we found three indirect paths: through body appreciation (Beta = 0.08, 95%CI = [0.02, 0.16]), through compromised masculine identity (Beta = 0.52, 95%CI = [0.34, 0.70]), and through body appreciation followed by compromised masculine identity (Beta = 0.09, 95%CI = [0.04, 0.15]).

### 3.2. Hypothesis 2: Among Men with FMS

Age at symptom onset and diagnosis were not correlated with psychological distress; therefore, no covariates were included in subsequent models (Table 5).

- Hypothesis 2a

As hypothesized, among men with FMS, PD was positively related to pain, illness-related shame and sense of compromised masculinity (Table 6). However, in contrast to our hypothesis, body appreciation was not correlated with pain and PD.

- Hypothesis 2b

**Figure 4:**
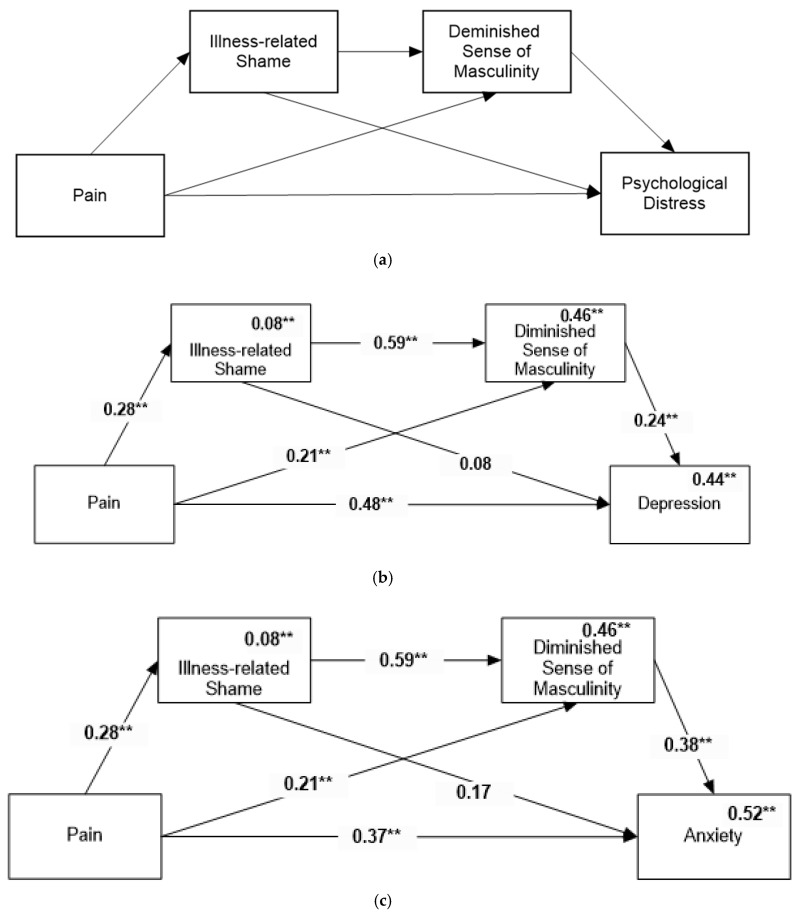
Revised mediation model for men with FMS (**a**). Mediation model for predicting depression among men with (**b**). Mediation model for predicting anxiety among men with FMS (**c**). Numbers above the lines are standardized path coefficients. Numbers above variable names are multiple squared correlations. ** *p* < 0.01.

As there was no association between body appreciation and either PD or pain, body appreciation was removed from the mediation model. The revised model is presented in Figure 4a.

The model predicting depression (Figure 4b) revealed, in addition to the direct path, two indirect paths: through compromised masculine identity (Beta = 0.05, 95%CI = [0.004, 0.11]) and through shame followed by compromised masculine identity (Beta = 0.04, 95%CI = [0.005, 0.09]).

Similarly, the model predicting anxiety (Figure 4c) revealed, in addition to the direct path, identical indirect paths: through compromised masculine identity (Beta = 0.05, 95%CI = [0.004, 0.11]) and through shame followed by compromised masculine identity (Beta = 0.04, 95%CI = [0.005, 0.09]).

### 3.3. Additional Findings

**Figure 5:**
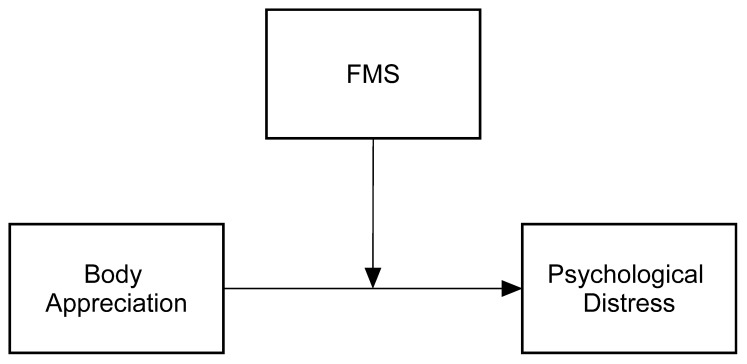
The proposed moderating role of health status in the link between body appreciation and PD.

The lack of correlation between body appreciation and PD, contrary to the literature identifying it as a protective factor, prompted us to examine whether its role differs between men with FMS and the control group. This was tested using the moderation model shown in Figure 5.

A significant interaction emerged between FMS and body appreciation in predicting depression. Among controls, body appreciation correlated negatively with depression (r = −0.48, *p* < 0.001), whereas no significant association was found in the FMS group (r = −0.18, ns). A similar, though non-significant, pattern was observed for anxiety. These results suggest that body appreciation does not operate as a protective factor among FMS patients, consistent with their elevated levels of PD.

## 4. Discussion

Fibromyalgia is more prevalent in women, and psychological research has therefore focused largely on women [6]. This study addresses the resulting gap by examining gender-specific mechanisms of distress in men with FMS. Results showed that men with fibromyalgia reported significantly higher depression and anxiety, lower body appreciation, and reduced masculine identity. These findings align with prior evidence of elevated distress in this population [10,11] and may be further exacerbated by the long delay between symptom onset and diagnosis, which in our sample averaged 4–5 years and may contribute to the “double burden of credibility” [6]. The present findings further highlight body image and masculine identity as key vulnerability factors. Mediation analysis indicated that body appreciation mediates the association between fibromyalgia and psychological distress, echoing findings in women [13,14]. This suggests that body appreciation may operate as a broader mechanism influencing mental health in fibromyalgia, although the absence of its protective role among men with FMS, discussed below, warrants cautious interpretation.

Masculine identity emerged as a key mechanism in both mediation models. When comparing men with FMS to HPs, reduced body appreciation predicted weakened masculine identity, which in turn heightened depression and anxiety. This aligns with evidence that chronic pain and functional limits prompt ongoing renegotiation of identity [12] and reduced body appreciation in FMS [13]. It thus may be assumed that participants’ loss of physical capability threatens hegemonic masculine norms, prompts ongoing identity renegotiation and increases psychological distress. In the FMS-only model, illness-related shame and compromised masculinity were the strongest pathways to psychological distress, with body appreciation no longer contributing. This pattern reflects the central role of shame in chronic illness, where loss of autonomy and a sense of bodily failure frequently lead to depression and anxiety [34]. Threats to masculine identity organized around strength, competence, and independence further amplify distress [25], and qualitative studies illustrate how these pressures interact with internalized stigma and role performance shame [6,12]. These dynamics are embedded in cultural contexts in which traditional masculine norms that emphasize physical capability, protection, and breadwinning remain highly salient [47,48]. Such expectations amplify stigma, inhibit symptom disclosure, discourage help seeking, and pressure men to persevere despite pain, thereby exacerbating distress [7,29].

The contrast between intra-group and inter-group analyses poses a theoretical challenge. Among HPs, higher body appreciation was strongly linked to better psychological wellbeing, but this association was absent in men with FMS. Moderation analysis indicated that body appreciation failed to buffer PD in men with FMS, contradicting evidence that it generally serves as a protective factor across chronic conditions [17]. Fibromyalgia’s invisible yet pervasive symptoms may disrupt the link between body appreciation and psychological wellbeing by fostering experiences of the body as unreliable or alien [12]. This disruption is reinforced by the well-documented cycle linking pain and distress in FMS: pain impairs functioning and social roles, heightening depression and anxiety, while distress intensifies pain through hypervigilance and catastrophizing [49,50]. Within this cycle, any protective effect of body appreciation may be limited [51]. Several factors may account for the missing effect. The high and persistent symptom burden of FMS can exceed the buffering capacity of psychological resources [51,52], while the unpredictability of symptoms may suppress body appreciation to a level that creates a floor effect [53]. Measurement issues may also play a role, as common body appreciation scales underrepresent bodily trust and functionality, which may be especially relevant for men with FMS. In addition, restricted variability in depression and anxiety scores may further reduce observable correlations [51].

### Limitations

The findings should be interpreted considering several limitations. First, the cross-sectional design prevents causal inference. Second, illness status was self-reported rather than clinically verified; variation in diagnostic source and criteria may have increased misclassification and heterogeneity in the fibromyalgia group due to misunderstanding, recall error, or symptom overlap, potentially biasing observed associations. Third, online recruitment may have introduced selection bias, as individuals in support groups often report greater illness burden [54]. Fourth, the sample included only Israeli participants. Fifth, comorbid chronic conditions were not assessed and may have influenced the findings. Sixth, 31% of participants did not complete the survey. Seventh, future studies may benefit from including the Functionality Appreciation Scale (FAS) [55], as the BAS-2 may be susceptible to floor effects in chronic-pain populations. Finally, the study focused on a limited set of mediators, leaving other relevant psychological and social mechanisms, such as illness perceptions, coping style, and social support, for future research.

Despite these limitations, the findings offer clear implications for practice. Clinical care should address identity and role disruption alongside symptom management, helping men develop a broader and more flexible sense of masculinity in which “strength” includes persistence, timely help seeking, caregiving, and values-based action, rather than forms of vulnerability that conflict with internalized norms [56]. Body-focused interventions should prioritize functionality, confidence in safe movement, pacing, and interoceptive awareness and trust rather than appearance-based acceptance [57,58], and may be strengthened by acceptance- and commitment-based approaches that explicitly target shame and values guided action [59]. A multidisciplinary approach that combines management of pain, sleep, and mood with gender-sensitive psychoeducation and body-oriented methods may reduce shame, lessen identity threat, increase engagement, and improve psychological wellbeing [7,12]. During assessment, clinicians can complement the standard pain history by asking, “Beyond the pain itself, what bothers you most about having fibromyalgia?” or “How has this affected the role you mentioned?” to identify shame, avoidance, and identity threats that may maintain distress.

Overall, this study deepens understanding of how fibromyalgia contributes to psychological distress in men by highlighting the central role of identity and social role disruption. The findings underscore the need for gender-sensitive models of chronic pain that recognize how illness differently shapes masculine and feminine identity. Future research should further examine the interplay between functionality and functionality appreciation to support the development of more tailored and effective psychological interventions.

## Figures and Tables

**Table 1:** Sample demographics and group comparison of background variables.

	FMS (N = 102)		Control (N = 123)		F(1, 223)	ᵡ^2^(1)
	M (SD)	N (%)	M (SD)	N (%)		
Age	39.8 (9.3)		33.3 (10.0)		25.2 **	
Years of Schooling	13.7 (2.6)		15.1 (3.0)		14.1 **	
Number of Children	1.7 (1.7)		1.3 (1.7)		3.7	
In a Relationship (yes)		63 (62)		95 (77)		5.9 *

* *p* < 0.05, ** *p* < 0.01.

**Table 2:** Correlations between the background characteristics and the outcome variables.

	Depression	Anxiety
Age	0.27 **	0.29 **
Years of Schooling	−0.29 **	−0.22 **
Number of Children	0.17 **	0.20 **
In a Relationship (yes)	0.18 **	0.06

** *p* < 0.01.

**Table 3:** Group comparison in the main study variables. Numbers in the cells are M (SD).

	FMS (N = 102)	Control (N = 123)	F(1, 223)
Depression	28.0 (5.2)	15.8 (4.7)	345.1 **
Anxiety	21.5 (5.1)	12.2 (4.1)	232.4 **
Body Appreciation	3.1 (0.9)	3.7 (0.9)	33.9 **
Compromised Masculine Identity	3.3 (1.0)	1.6 (0.7)	234.2 **

** *p* < 0.01.

**Table 4:** Correlations between the main study variables.

	Depression	Anxiety	Body Appreciation
Anxiety	0.82 **		
Body Appreciation	−0.48 **	−0.41 **	
Compromised Masculine Identity	0.79 **	0.79 **	−0.50 **

** *p* < 0.01.

**Table 5:** Descriptive statistics for illness-related variables (N = 102).

	M (SD)	Range
Pain	16.2 (15.3)	0.0–45.0
Illness-related Shame	3.3 (1.1)	1.0–5.0
Age at Symptom Onset	31.4 (9.5)	14.0–56.0
Age at Diagnosis	35.9 (9.9)	16.0–63.0

**Table 6:** Correlations between the main study variables and illness-related variables among men with FMS.

	1	2	3	4	5
Depression					
Anxiety	0.23 **				
Body Appreciation	−0.18	−0.13			
Compromised Masculine Identity	0.48 **	0.62 **	−0.31 **		
Illness-related Shame	0.34 **	0.52 **	−0.24 *	0.65 **	
Pain	0.60 **	0.55 **	0.18	0.38 **	0.28 **

* *p* < 0.05, ** *p* < 0.01.

## Data Availability

The data presented in this study are available on request from the corresponding author. The data are not publicly available due to ethical restrictions.

## Abbreviations

The following abbreviations are used in this manuscript:

FMS	Fibromyalgia syndrome
HPs	Healthy peers
